# The Association Between Pre-Pregnancy and First-Trimester Hair Cortisol and Preterm Birth: A Causal Inference Model

**DOI:** 10.21203/rs.3.rs-4095921/v1

**Published:** 2024-04-25

**Authors:** Yinxian Chen, Richard G. Künzel, Sixto E. Sanchez, Marta B. Rondon, Nelida I. Pinto, Elena Sanchez, Clemens Kirschbaum, Linda Valeri, Karestan C. Koenen, Bizu Gelaye

## Abstract

Background Adverse life events and chronic psychological distress before and during pregnancy have frequently been associated with preterm birth (PTB) but the biological underpinnings remain unclear. We investigated the association between corticosteroid levels in pre-pregnancy and first-trimester hair and the risk of PTB. Methods We followed 1,808 pregnant women from a prospective pre-birth cohort study in Lima, Perú. Hair samples were taken at the end of the first pregnancy trimester. The two most proximal 3cm segments to the scalp (representing pre-pregnancy and first-trimester) were analyzed to obtain hair cortisol and cortisone concentrations (HCC and HCNC). PTB was defined as birth < 37 completed gestational weeks. We constructed four generalized propensity scores for pre-pregnancy and first-trimester HCC and HCNC to create corresponding inverse probability weights before fitting marginal structural models for estimating the effect of HCC and HCNC on PTB risk. Results Pre-pregnancy Log HCC was not independently associated with PTB risk (RR = 0.97; 95%CI: 0.79, 1.19). In contrast, one SD increase from the mean first-trimester Log HCC was independently associated with a 37% (95%CI: 1.11, 1.69) increased risk of PTB. Although imprecise, pre-pregnancy Log HCNC was negatively associated with PTB risk (RR = 0.84; 95%CI: 0.58, 1.20), whereas the association between first-trimester Log HCNC and PTB risk was positive (RR = 1.20; 95%CI: 0.87, 1.65). Conclusions Our findings show that chronic corticosteroid levels in early pregnancy are causally linked to PTB risk in pregnant Peruvian women. This finding contributes to understanding the biological underpinnings of PTB better to enhance PTB prevention.

### 2 **Introduction**

Preterm birth (PTB) remains one of the most pressing and unsolved issues in public health [1], accounting for 18-28 % of childhood mortality until the age of 5 years [2,3]. Despite improvements in PTB prevention [4], approximately 13 million children are born prematurely worldwide every year [1] with disproportionally higher rates in low and middle-income countries compared to high-income countries [1].

PTB can have severe life-course consequences for both mother and child. For example, children born preterm face an increased risk for cardiovascular and renal disease [5], diabetes [6], asthma [7] and neurodevelopmental impairment [8], among other conditions [9]. Accordingly, women who deliver preterm have a higher risk for cardiovascular disease than those who deliver at term [10]. Likewise, PTB has been associated with an elevated risk for maternal psychopathology including postpartum depression [11,12]. Although the specific etiology of PTB remains unknown, an increased risk for PTB has been associated with family and obstetric history of PTB [13,14], maternal pre-pregnancy health status [15,16], but also social and structural disparities, such as racism and historical redlining [17,18], among others [19]. Furthermore, the effect of psychological distress on PTB is a rapidly evolving area of investigation as a number of studies, including our own, have found associations between symptoms of mood [20–25] and anxiety disorders [23,24,26–29] and elevated risk for PTB, although not consistently [26,30,31]. Additionally, previous research has identified psychologically distressing events, such as early life adversity or intimate partner violence, as risk factors for PTB [32–34], that often precede symptoms of psychological distress [35,36].

The specific biological underpinnings between symptoms of psychological distress and PTB risk also remain unknown. Nevertheless, the hypothalamic-pituitary-adrenal (HPA) axis has been suggested as a possible biological pathway, as it is the body's primary stress-response system

[37]. In response to stressful experiences, the hypothalamus gets stimulated, secreting the corticotropin-releasing hormone (CRH) [38,39]. CRH acts stimulating on the pituitary, that secretes the adrenocorticotropic hormone (ACTH) into the blood stream, which targets the adrenal cortex [40]. The adrenal cortex then secrets glucocorticoids and catecholamines [40], of which cortisol and cortisone are the most important for HPA axis functioning [41]. Interestingly, cortisol is also involved in pregnancy regulation, as findings of increasing average cortisol concentrations across pregnancy, measured with varying biospecimen, suggest [42,43]. Human and animal studies indicate that cortisol and its related hormones are involved in labor onset [44], as well as fetal brain and lung development [45,46]. Cortisol and cortisone can be measured in urine, blood and saliva, although situational and diurnal variations limit their use for long-term evaluation of HPA axis activity [47]. Therefore, cortisol and cortisone assessments in human scalp hair have emerged as novel tools to assess long-term hormone secretion in recent years [48]. Because hair growth is relatively stable over time [49,50], hair cortisol concentration (HCC) and hair cortisone concentration (HCNC) indicate the respective hormone secretion over a specific timespan [51], such as pregnancy. Hence, HCC and HCNC have become promising biomarkers of psychological distress in both, pregnant and non-pregnant individuals [52,53] and respective correlations with PTB have been shown in previous studies [28,54–60].

However, previous studies have solely conducted correlational analyses predominantly with samples from high income countries, that yielded inconclusive results regarding the predictive value of HCC and HCNC for PTB, albeit the urgent need to identify the causal role chronic cortisol and cortisone play in PTB etiology. Furthermore, previous research noted that baseline cortisol levels can be chronically altered depending on a history of traumatic experiences, raising the question of whether such pre- and early pregnancy HCC alterations could impact the pregnancy course and outcomes [61].

Therefore, in the present study we aimed to i) identify the independent causal relationship between HCC, HCNC and PTB risk and ii), investigate the cumulative effect of HCC and HCNC at different pregnancy time points on PTB risk in a large sample of an understudied population.

## 3 **Methods**

### 3.1 Study Population

The sample for this study was drawn from participants of the Pregnancy Outcomes, Maternal, and Infant Study (PrOMIS), a prospective cohort study with the objective to identify maternal, social and behavioral risk factors for adverse pregnancy outcomes in Lima, Perú. Participants were women attending prenatal care clinics of the Instituto Nacional Materno Perinatal (INMP), which serves as the principal establishment for maternal and prenatal care in Perú. Study methodology has been reported previously [62]. Participants were recruited between February 2012 and November 2015 and followed up until delivery. Participants were eligible if they initiated prenatal care prior to the 16th gestational week [62], were ≥ 18 years old, were able to speak and read Spanish, had a singleton pregnancy, and intended to deliver at an INMP. Written informed consent was obtained from all participants. All study procedures were approved by the institutional review boards of the INMP, Lima, Perú and the Harvard T.H. Chan School of Public Health, Boston, MA, USA. In this study, participants with two measures in at least one type of hair corticosteroid concentration and a record of gestational age at birth were included in the study population.

### 3.2 Data Collection

#### *3.2.1 Sociodemographic, reproductive and covariate data*

Sociodemographic, medical and reproductive history and hair-related data were collected through structured interviews conducted by trained research personnel at the time of recruitment. The socio-demographic data included maternal age (years), marital status (married or live with partner/ other), ethnicity (mestizo/ non-mestizo), employment during pregnancy (yes/ no) and difficulty paying for basic items such as food (yes/ no). Pre-pregnancy and first-trimester weight (kg) and height (m) were collected to the nearest 0.1 kg and 0.1 cm to calculate the respective body mass index (BMI; $kg/m^2$). Gestational age upon registration was determined by self-report of the last menstrual period and confirmation by ultrasound examination prior to 20 weeks of gestation. Hair-related information at data collection included hair washing frequency (1-2 times/week, 3-5 times/week, 6–7 times/week), hair dye (yes/ no), and hair tint (yes/ no). Medical and reproductive history information included planned pregnancy (yes/ no), parity (nulli-/ multiparous) and infant sex (male/ female). After delivery, medical records of the mother and newborn were abstracted to assess the pregnancy course and outcomes, including PTB. We defined PTB as delivery prior to completed gestational week 37, according to guidelines by the American College of Obstetricians and Gynecologists (ACOG) [63] and categorized PTB cases according to three pathophysiological groups (spontaneous PTB, preterm premature rupture of membranes, and medically induced PTB). Spontaneous PTB cases comprised women with a physician diagnosis of spontaneous labor onset (with intact fetal membranes) and delivery prior to the completion of 37 weeks' gestation, as indicated by medical records. Preterm premature rupture of membranes cases comprised women with a physician diagnosis of rupture of fetal membranes (prior to the onset of labor) and delivery prior to the completion of 37 weeks' gestation, as recorded in medical records. Women who delivered before completed gestational week 37 due to medical intervention were ineligible for this study

*3.2.2 Hair Collection Procedures*

Full-length hair samples were collected by trained research personnel from the posterior vertex region of the head at the end of the first pregnancy trimester. Hair was cut as close to the scalp as possible and cut into three 3 cm segments. Thus, each segment reflected approximately 3 months, based on an average growth rate of 1 cm/month [49,50]. Only the two segments closest to the scalp were analyzed due to possible washout effect, resulting in less reliable measurements of cortisol in the most distal hair segment [51,64]. Hence, the proximal 3 cm segment reflected the first trimester of pregnancy, and the distal segment reflected 0-3 months pre-pregnancy. After collection, hair samples were wrapped in aluminum foil, stored in envelopes away from light, and kept at room temperature using desiccants. Hair samples were sent to the Kirschbaum laboratory at Dresden University of Technology, Germany, for analysis.

##### *3.2.3 Laboratory Analysis*

First, hair samples were washed twice in 2.5 ml isopropanol for three minutes in a 15-ml falcon tube and dried for 12 hours. Second, 7.5 mg of whole non-pulverized hair was weighed out per segment and minced into small pieces. This procedure was chosen according to in-house experiment results by the executing lab [65]. Third, at room temperature, hair samples were incubated in 1.8 ml of high-grade methanol for 18 hours. Fourth, 1.6 ml of clear supernatant was transferred to a vial. Fifth, the methanol was evaporated off for 30 minutes at 55 °C using nitrogen and then resuspended in 225 µl distilled water before adding 20 µl internal standard (cortisol-d4). Sixth, HCC and HCNC levels were quantified by Cortisol Luminescence Immunoassay (IBL International ®, Hamburg, Germany) using 50 µl of the total resuspension. The immunoassay had a lower detection limit of 0.1 pg/mg and an intra- and inter-assay coefficient of variation of 11.9% and 19.4%, respectively, within acceptable ranges. Hair segments from the same participant were assayed together in the same batch to minimize batch-to-batch variability. HCC and HCNC units were expressed in picograms per milligram (pg/mg).

#### 3.3 Statistical Analysis

Descriptive statistics, where mean (SD) was for continuous variables and number (%) for categorical variables, were displayed for characteristics of the study population. The mean (SD) and median (IQR) were also summarized for HCC and HCNC in pre-pregnancy and first trimester.

Before we performed the causal association assessment with full measures of HCC or HCNC, we trimmed each sub-population at 1 and 99 percentiles of HCC or HCNC to minimize the impact of extreme values on estimating the association. Since the missing rate in each variable was <5% (Table S1), the primary analysis was focused on samples with complete data on all included covariates (i.e., the complete case analysis). Pre-pregnancy and first trimester HCC and HCNC were transformed into the natural log scale due to their skew distributions in the population before being standardized to mean = 0 and SD = 1. First, a total of four generalized propensity scores (GPS) were constructed for pre-pregnancy and first trimester HCC and HCNC using the generalized additive model (GAM). Each model contained penalized regression spline terms for all continuous predictors to account for their potential non-linear association with hair corticosteroid concentration. The covariates for each propensity score model were selected if they were potential confounders based on their hypothetical association with hair corticosteroid concentration and PTB (Fig.S1). Second, we utilized stabilized inverse-probability weights (SIPW) calculated by GPS to construct marginal structural models (MSM) using Poisson regression to estimate the independent causal association of HCC and HCNC on two occasions with risk of PTB, respectively (Table S2). We reported relative risks (RRs) for each occasion and 95% confidence intervals (CIs) calculated by robust standard errors. The cumulative association of HCC and HCNC across time with PTB risk could then be calculated by multiplying RRs on two occasions. To examine whether the SIPW created by GAM-estimated GPS was successful in addressing confounding, we compared the unweighted correlation between hair corticosteroid concentration and each potential confounder with the correlation weighted by SIPW. We computed the average absolute correlation (AAC) with and without SIPW to display the overall performance of achieving covariate balance and balance plots to illustrate the performance for each covariate. The method for calculating the correlation has been previously described. [66,67] Secondary analyses were focused on: (1) testing whether the cumulative association of HCC and HCNC with PTB risk would varied depending on the levels of HCC and HCNC at different times by comparing MSM with and without the product term "Pre-pregnancy*First trimester", and (2) testing the potential curvilinearity in the association of HCC and HCNC with PTB risk by fitting MSM using GAM with penalized spline terms and comparing with the original MSM in primary analysis. All tests were performed by the Likelihood ratio test.

Several sensitivity analyses were performed. First, we addressed the missing values by employing multiple imputation using the *mice* package in R (Appendix S1) and reran the primary analysis. Second, we fit the GPS using multivariate linear regression instead to create SIPW. We also compared the weighted AAC, as well as balance plots, by different GPS fitting approaches (i.e., GAM vs. linear regression).

## 4 Results

### 4.1 Descriptive Results

This study included *N* = 1,808 pregnant women, with the demographic characteristics and outcome distribution described in Table 1. The mean age of participants was 28.0 (SD = 6.2) years. The majority of participants reported being married or living with a partner (83 %), being of mestizo ethnicity (80%) and being pregnant unplanned (59%). Approximately half of the participants reported employment during pregnancy (49 %), being nulliparous (47 %) and having difficulty paying for basics such as food (45 %). BMI did not substantially vary, with the mean BMI shifting from 25.5 (SD = 4.0) kg/$m^2$ before pregnancy to 25.7 (SD = 4.1) kg/$m^2$ at the recruitment visit during pregnancy. Regarding hair treatment, most of the women (71 %) reported a hair wash 3-5 times/ week, and hair tint (43 %) was more common than hair dye (15 %). The mean gestational age during the hair samples collection was 14.8 (SD = 7.5) weeks. The cumulative incidence of spontaneous PTB in this population was 7 % during the follow-up, and male infants accounted for 51 % of the total births.

**Table 1.** Characteristics of pregnant women sample. Lima, Perú.

| Characteristics | All participants (*N* = 1,808) |
|---|---|
| Maternal age (year), mean (SD) | 28.0 (6.2) |
| Maternal age (year) category | |
| 18-19 | 101 (5.6) |
| 20-29 | 1,009 (55.8) |
| 30-34 | 393 (21.7) |
| ≥35 | 304 (16.8) |
| Difficulty in paying for basics | |
| No | 988 (54.9) |
| Yes | 811 (45.1) |
| Employment during pregnancy | |
| Unemployed | 913 (50.6) |
| Employed | 893 (49.4) |
| Marital status | |
| Others | 311 (17.2) |
| Married or live with a partner | 1,492 (82.8) |
| Nulliparity | |
| No | 959 (53.2) |
| Yes | 843 (46.8) |
| Ethnicity | |
| Not Mestizo | 354 (19.6) |
| Mestizo | 1,451 (80.4) |
| Planned pregnancy | |
| Unplanned | 1,065 (59.3) |
| Planned | 732 (40.7) |
| Gestational age at hair collection, mean (SD) | 14.8 (7.5) |
| Infant sex | |
| Male | 909 (51.2) |
| Female | 867 (48.8) |
| Pre-pregnant BMI (kg/m$^2$), mean (SD) | 25.5 (4.0) |
| Pre-pregnant BMI category | |
| <18.5 | 26 (1.4) |
| 18.5-24.9 | 886 (49.3) |
| 25.0-29.9 | 657 (36.5) |
| ≥30 | 229 (12.7) |
| BMI at pregnancy (kg/m$^2$), mean (SD) | 25.7 (4.1) |
| BMI category at pregnancy | |
| <18.5 | 32 (1.8) |
| 18.5-24.9 | 820 (45.7) |
| 25.0-29.9 | 696 (38.8) |
| ≥30 | 246 (13.7) |
| Preterm birth (gestational age <37 weeks) | |
| No | 1,677 (92.8) |
| Yes | 131 (7.2) |
| Hair dye | |
| Yes | 261 (14.6) |
| No | 1,521 (85.4) |

| | |
|---|---|
| Hair tint | |
| Yes | 761 (42.7) |
| No | 1,022 (57.3) |
| Hair wash | |
| 1-2 times/week | 68 (3.8) |
| 3-5 times/week | 1,270 (71.2) |
| 6-7 times/week | 446 (25.0) |

Abbreviations: BMI, body mass index; SD, standard deviation

[a]The number of participants in each variable may not be identical to the total sample sizes due to the missingness.

Among the *N* = 1,808 individuals included in the study, *n* = 1,800 had data on HCC, *n* = 1,661 on HCNC. As described in Table 2, the HCC and HCNC increased in the first trimester of pregnancy compared with the pre-pregnancy levels. However, the increase was more pronounced in HCNC compared with HCC.

**Table 2.** Characteristics of hair corticosteroid levels/ratio of pregnant women during pre -pregnancy and first trimester of pregnancy. Lima, Perú.

| | **Hair segment** | |
|---|---|---|
| | Pre-pregnancy | First trimester |
| **HCC (pg/mg)** | *N* = 1,800 | |
| Mean (SD) | 4.12 (5.10) | 5.10 (6.54) |
| Median (IQR) | 2.83 (2.67) | 3.57 (3.26) |
| **HCNC (pg/mg)** | *N* = 1,661 | |
| Mean (SD) | 5.33 (5.85) | 9.95 (9.50) |
| Median (IQR) | 3.74 (3.69) | 7.38 (6.41) |

Abbreviations: HCC, hair cortisol concentration; HCNC, hair cortisone concentration; IQR, interquartile range; SD, standard deviation

### 4.2 Primary Analysis

Table 3 shows that one SD increase from the mean pre-pregnancy Log HCC was not associated with the risk of PTB (RR = 0.97, 95% CI: 0.79, 1.19). In contrast, one SD increase from the mean pre-pregnancy Log HCNC was independently associated with a 16% decreased risk of PTB, although imprecisely (95%CI: 0.58, 1.20).

Regarding the first trimester, a one SD increase from the mean first trimester Log HCC was independently associated with 37 % increased risk of PTB (95%CI: 1.11, 1.69). Correspondingly, a one SD increase in the first trimester Log HCNC was independently associated with 20% increased risk of PTB, although imprecisely (95%CI: 0.87, 1.65).

**Table 3.** Marginal association between hair corticosteroid levels and risk of preterm birth

| | Pre-pregnancy | First trimester |
|---|---|---|
| | RR (95%CI) | RR (95%CI) |
| Log HCC[a] (N = 1647) | 0.97 (0.79,1.19) | 1.37 (1.11,1.69) |
| Log HCNC[a] (N = 1520) | 0.84 (0.58,1.20) | 1.20 (0.87,1.65) |

Abbreviations: HCC, hair cortisol concentration; HCNC, hair cortisone concentration
[a] Log HCC and Log HCNC have been standardized (mean=0 and SD=1).

### 4.3 Secondary analysis

Table 4 shows that the cumulative association of Log HCC with PTB risk did not vary by the level of pre-pregnancy and first trimester Log HCC ($\beta$ = -0.01, $P$ = 0.89). Little evidence indicates that the cumulative association of Log HCNC with PTB risk varied by the level of pre-pregnancy and first trimester Log HCNC ($\beta$ = -0.12, $P$ for interaction = 0.20). Furthermore, little evidence shows that Log HCC and Log HCNC were associated with PTB risk in a non-linear pattern (Fig. S4, Fig. S5).

**Table 4.** Test for interaction between hair corticosteroid levels on different occasions in relation to risk of preterm birth

| | Pre-pregnancy | First trimester | Pre-pregnancy*First trimester | |
|---|---|---|---|---|
| | RR (95%CI) | RR (95%CI) | $\beta$ | $P$[b] |
| Log HCC ($N$ = 1647) | 0.97 (0.77,1.23) | 1.37 (1.12,1.68) | -0.01 | 0.89 |
| Log HCNC ($N$ = 1520) | 0.83 (0.58,1.19) | 1.19 (0.88,1.63) | -0.12 | 0.20 |

Abbreviation: HCC, hair cortisol concentration; HCNC, hair cortisone concentration.
[a] Log HCC and Log HCNC have been standardized (mean=0 and SD=1).
[b] $P$ was derived from the Likelihood-ratio test comparing the model with to without the interaction term (Pre pregnancy*First trimester)

### 4.4 Covariate balance

Table 5 shows that SIPW created by GAM-estimated GPS successfully made Log HCC and Log HCNC independent of potential confounders. Weighted AAC was substantially dropped compared with unweighted AAC in both Log HCC and Log HCNC across occasions. Fig. S2 and Fig. S3 show that SIPW shrank each absolute correlation between Log HCC, as well as between Log HCNC, and potential confounder to lower than 0.1, indicating that it had a satisfactory performance in achieving covariate balance.

### 4.5 Sensitivity analysis

Table S3 shows that estimates from the imputed analysis were highly consistent with the complete case analysis. By using linear regression to fit GPS and create SIPW, the association estimates were concordant with the estimates from using GAM (Table S4). The weighted AAC by linear regression and GAM were almost identical (Table S5), and the performance of shrinking each absolute correlation was similar (Fig. S6 and S7).

**Table 5.** Average absolute correlation between hair corticosteroid levels and each covariate without and with IPW

| | Pre-pregnancy | | First trimester | |
|---|---|---|---|---|
| | Unweighted AAC | Weighted AAC | Unweighted AAC | Weighted AAC |
| Log HCC (N = 1647) | 0.042 | 0.008 | 0.061 | 0.026 |
| Log HCNC (N = 1520) | 0.067 | 0.013 | 0.073 | 0.048 |

Abbreviation: AAC, average absolute correlation; HCC, hair cortisol concentration; HCNC, hair cortisone concentration.

### 5 Discussion

To shed further light on the causal role of chronic corticosteroids in PTB etiology, our study examined the effect of pre- and early pregnancy corticosteroid levels on PTB risk in a large sample of Peruvian women. We found that an independent increase in pre-pregnancy HCNC was imprecisely associated with decreased risk of PTB, whereas an independent increase of HCC and HCNC in the first trimester was associated with an increased PTB risk, although precise only for HCC.

Our finding of an independent, although small and imprecise, negative effect of pre-pregnancy corticosteroid levels increases on PTB risk is in line with our previous study of *N* = 137 pregnant women from Perú [55], showing that the increase in pre-pregnancy HCC was associated with decreased adjusted odds for PTB. Our study site Perú is a low and middle income country with high rates of unintended pregnancies (59-65 %) [68] and traumatic life events, such as intimate partner violence (40%) [68,69] and childhood abuse (72 %) [70]. Previous research has implied that traumatic life events can alter long-term HPA axis functioning in a time- and dose-dependent manner [61]. More specifically, Steudte-Schmiedgen et al. [61] suggested that repeated experiences of trauma may result in an attenuated long-term cortisol concentration. Given that more than 50% of study participants reported experiences of traumatic life events, we suppose that a *mean* corticosteroid level in our sample actually represents a *low* level compared to other samples, possibly due to a trauma related chronic HPA axis alteration. For instance, the first-trimester HCC level observed in our study is, on average lower than values reported in comparable studies, as indicated by findings of a recent systematic review by Marceau et al. reviewing 56 studies from diverse samples [71]. Unfortunately, no comparison to pre-pregnancy corticosteroid levels in other samples can be drawn, because to date no other studies with that scope exist. Nevertheless, we suppose that the protective effect of pre-pregnancy HCNC level increases on PTB risk as found in our study is driven by a high prevalence of stress-related chronically low pre-pregnancy corticosteroid levels in our sample. However, we did not find lower corticosteroid levels among participants with a history of adverse life events in our previous study [72]. This might be due to a missing consideration of the mental exposure to traumatic stressors at data assessment as Steudte-Schmiedgen et al. [61] explained previous findings of Schalinski et al.[73] (reporting elevated HCC levels among traumatized participants) with the fact that most of the participants were still exposed to trauma-related stressors. This potential associational mechanism given, any biologic conclusions drawn from our findings must be taken cautiously, given the small and imprecise nature of the pre-pregnancy effects.

In contrast, we found an elevated risk of PTB for increases in the first-trimester corticosteroid level. This is in accordance with results by Hoffman et al. [56], who found a negative correlation between the first-trimester HCC and gestational age at delivery among $N = 90$ healthy pregnant US-American women [56]. Previous research reports associations between stressor-specific acute stress-response alterations and the experience of traumatic life events [41,74–78]. For example, traumatized individuals showed an attenuated cortisol response to laboratory social stress inductions compared to non-traumatized individuals [74–76] but exposure to a trauma related-condition, such as trauma recall or confrontation, was associated with an elevated cortisol response [77,78]. Almost 60 % of the participants of our sample reported an unplanned pregnancy, which may potentially serve as a stressful abuse- or violence-related condition. Given that over 70% of the participants reported child abuse and 30% IPV, we suppose that our finding of an increased PTB risk for the first-trimester corticosteroid increases is driven by an increased pregnancy-related stress response among traumatized individuals. In contrast, other studies suggest a positive association between the first-trimester HCC and gestational age [28,58,59]. For example, our previous study found a 61 % decreased adjusted odds of PTB for every one-unit increase of the first-trimester HCC, albeit not statistically significant [55]. A significant association between HCNC and gestational length was neither found by Musana et al. [58] in their study of $N = 130$ pregnant women in Kenya. However, for women of elevated pregnancy-related medical risk, there was a significantly higher HCNC in preterm than term-delivering women [58]. The heterogeny of the study populations, such as Canada [28], Israel [59] or Kenya [58], as well as low statistical power in previous studies, may contribute to inconsistency between other studies and our results. Additionally previous studies have not considered the cumulative effect of corticosteroid secretion at different pregnancy time-points in relation to PTB.

When examining this cumulative effect of chronic corticosteroids on PTB risk, we found a negative, although small and imprecise, cumulative effect for HCNC. That is, an increase in corticosteroid levels at only one occasion may yield a higher PTB risk than corticosteroid increases at both or neither occasion. According to our previous interpretation of the independent effects of corticosteroid increases on PTB risk, it could be inferred that chronically lower corticosteroid levels before pregnancy followed by a pregnancy-stress-related first-trimester corticosteroid increase could represent an abrupt, and hence, rather suboptimal adaption possibly leading to elevated PTB risk. However, the small and imprecise interaction limit such mechanistic inferences significantly but support our finding of the detrimental effect of independent first trimester corticosteroid increases on PTB risk to a greater extend.

Drawing on the contradictory findings of previous studies, finding both, higher [56,58] and lower corticosteroids [28,55,57] in relation to elevated PTB risk, we investigated potential non-linear associations between corticosteroid levels and PTB risk. Indeed, we found tentative evidence for a non-linear linkage. However, further studies are needed to allow for biological interpretations of this finding.

### 5.1 Limitations

The results of our study provide new insights into the causal effect of temporal chronic corticosteroid levels on PTB risk, implicating that the corticosteroid level an individual has at first trimester may significantly shape the pregnancy course and its outcomes. However, there are some limitations to consider.

First, under the current causal inference methodology, potential misspecification of the models used for creating the propensity scores and subsequent IPWs cannot be ruled out. Additionally, the assumption of no unmeasured confounders in this study might be challenged since this is an observational study with residual confounding that cannot be accounted for. Nevertheless, we included as many covariates as possible that might confound the causal effect of corticosteroid levels on PTB risk to try to eliminate the influence of residual confounding. Second, although we used the multiple imputations technique to adjust for the underlying bias caused by missing at random (MAR), the bias by potential missing not at random (MNAR) could not be addressed. However, since the total missing rate in this study is <5 %, the influence of MNAR seems unlikely. Third, our findings may not be generalizable to other populations. Although investigating pregnant women in Perú contributed to mitigating the imbalance between studies in high versus low- and middle-income countries, the particularly high exposure of study participants to life stressors such as poverty, household violence and abuse makes our study results unique.

Finally, our study scope was limited to corticosteroid assessments to pre-pregnancy and first trimester as we intended to examine the effect of corticosteroid levels around the time of conception on pregnancy outcomes. Future studies are needed to validate our findings and to expand this scope to the remaining pregnancy course.

### 6 Conclusion

Being born too soon can detrimentally affect health throughout the life course. To mitigate this detrimental effect, an understanding of the causal underpinnings of PTB is crucial. In our study of a large sample of pregnant women from Perú, we found that higher chronic corticosteroid levels in the first pregnancy trimester were associated with an increased risk of PTB, whereas higher levels during pre-pregnancy seem not to influence PTB risk substantially. Furthermore, non-linearity of this association and sample-specific cumulative effects of corticosteroid levels at different time-points seem possible. This linkage is a promising pathway to better understanding the pathogenesis of PTB.

### 7 Statements and Declarations

#### 7.1 Author contributions

BG conceived and designed the study. SES, ES and NIP led the field data collection. YC conducted the data analysis. RK and YC drafted the manuscript. YC, RGK, SES, MR, NIP, ES, CK, LV, KCK, and BG interpreted the results and critically revised the draft for important intellectual content. All authors read the manuscript and approved its last version for publication.

#### 7.2 Funding

The PrOMIS study was supported by awards from the National Institutes of Health (NIH), the Eunice Kennedy Shriver National Institute of Child Health and Human Development (R01HD102342 and R21HD102822), and National Institutes of Mental Health (R21MH128985). The funder had no further involvement in the study design, analysis, and interpretation of evidence, not in the manuscript preparation and decision to submit the paper for publication.

#### 7.3 Declaration of Competing Interest

The authors declare no competing interests.

#### 7.4 Consent to Participate

Written informed consent was obtained from all participants.

#### 7.5 Ethics Approval

All study procedures used in this research were approved by the institutional review boards of the Instituto Nacional Materno Perinatal, Lima, Peru and the Harvard T.H. Chan School of Public Health, Boston, MA, USA.

## References

1. Ohuma, E. O. *et al.* National, regional, and global estimates of preterm birth in 2020, with trends from 2010: a systematic analysis. *The Lancet* **402**, 1261–1271 (2023).
2. Lawn, J. E., Wilczynska-Ketende, K. & Cousens, S. N. Estimating the causes of 4 million neonatal deaths in the year 2000. *Int. J. Epidemiol.* **35**, 706–718 (2006).
3. Perin, J. *et al.* Global, regional, and national causes of under-5 mortality in 2000–19: an updated systematic analysis with implications for the Sustainable Development Goals. *Lancet Child Adolesc. Health* **6**, 106–115 (2022).
4. Romero, R., Dey, S. K. & Fisher, S. J. Preterm labor: one syndrome, many causes. *Science* **345**, 760–765 (2014).
5. Abitbol, C. L. & Rodriguez, M. M. The long-term renal and cardiovascular consequences of prematurity. *Nat. Rev. Nephrol.* **8**, 265–274 (2012).
6. Li, S. *et al.* Preterm birth and risk of type 1 and type 2 diabetes: systematic review and meta-analysis. *Obes. Rev. Off. J. Int. Assoc. Study Obes.* **15**, 804–811 (2014).
7. Sonnenschein-van der Voort, A. M. M. *et al.* Preterm birth, infant weight gain, and childhood asthma risk: a meta-analysis of 147,000 European children. *J. Allergy Clin. Immunol.* **133**, 1317–1329 (2014).
8. Hee Chung, E., Chou, J. & Brown, K. A. Neurodevelopmental outcomes of preterm infants: a recent literature review. *Transl. Pediatr.* **9**, S3–S8 (2020).
9. Frey, H. A. & Klebanoff, M. A. The epidemiology, etiology, and costs of preterm birth. *Semin. Fetal. Neonatal Med.* **21**, 68–73 (2016).
10. Robbins, C. L., Hutchings, Y., Dietz, P. M., Kuklina, E. V. & Callaghan, W. M. History of preterm birth and subsequent cardiovascular disease: a systematic review. *Am. J. Obstet. Gynecol.* **210**, 285–297 (2014).

11. de Paula Eduardo, J. A. F., de Rezende, M. G., Menezes, P. R. & Del-Ben, C. M. Preterm birth as a risk factor for postpartum depression: A systematic review and meta-analysis. *J. Affect. Disord.* **259**, 392–403 (2019).

12. Trumello, C. *et al.* Mothers' Depression, Anxiety, and Mental Representations After Preterm Birth: A Study During the Infant's Hospitalization in a Neonatal Intensive Care Unit. *Front. Public Health* **6**, 359 (2018).

13. Kazemier, B. *et al.* Impact of obstetric history on the risk of spontaneous preterm birth in singleton and multiple pregnancies: a systematic review. *BJOG Int. J. Obstet. Gynaecol.* **121**, 1197–1208 (2014).

14. Koire, A., Chu, D. M. & Aagaard, K. Family history is a predictor of current preterm birth. *Am. J. Obstet. Gynecol. MFM* **3**, 100277 (2021).

15. Haas, J. S. *et al.* Prepregnancy Health Status and the Risk of Preterm Delivery. *Arch. Pediatr. Adolesc. Med.* **159**, 58–63 (2005).

16. Liu, B. *et al.* Association between maternal pre-pregnancy obesity and preterm birth according to maternal age and race or ethnicity: a population-based study. *Lancet Diabetes Endocrinol.* **7**, 707–714 (2019).

17. Krieger, N., Huynh, M., Li, W., Waterman, P. D. & Van Wye, G. Severe sociopolitical stressors and preterm births in New York City: 1 September 2015 to 31 August 2017. *J. Epidemiol. Community Health* **72**, 1147–1152 (2018).

18. Krieger, N. *et al.* Structural Racism, Historical Redlining, and Risk of Preterm Birth in New York City, 2013-2017. *Am. J. Public Health* **110**, 1046–1053 (2020).

19. *Preterm Birth: Causes, Consequences, and Prevention*. (National Academies Press (US), 2007).

20. Bansil, P. *et al.* Maternal and fetal outcomes among women with depression. *J. Womens Health 2002* **19**, 329–334 (2010).

21. Grote, N. K. *et al.* A meta-analysis of depression during pregnancy and the risk of preterm birth, low birth weight, and intrauterine growth restriction. *Arch. Gen. Psychiatry* **67**, 1012–1024 (2010).

22. Liu, C., Cnattingius, S., Bergström, M., Östberg, V. & Hjern, A. Prenatal parental depression and preterm birth: a national cohort study. *BJOG Int. J. Obstet. Gynaecol.* **123**, 1973–1982 (2016).

23. Sanchez, S. E. *et al.* Risk of Spontaneous Preterm Birth in Relation to Maternal Depressive, Anxiety and Stress Symptoms. (2014).

24. Staneva, A., Bogossian, F., Pritchard, M. & Wittkowski, A. The effects of maternal depression, anxiety, and perceived stress during pregnancy on preterm birth: A systematic review. *Women Birth* **28**, 179–193 (2015).

25. Straub, H., Adams, M., Kim, J. J. & Silver, R. K. Antenatal depressive symptoms increase the likelihood of preterm birth. *Am. J. Obstet. Gynecol.* **207**, 329.e1-329.e4 (2012).

26. Gelaye, B. *et al.* Association of antepartum depression, generalized anxiety, and posttraumatic stress disorder with infant birth weight and gestational age at delivery. *J. Affect. Disord.* **262**, 310–316 (2020).

27. Glynn, L. M., Schetter, C. D., Hobel, C. J. & Sandman, C. A. Pattern of perceived stress and anxiety in pregnancy predicts preterm birth. *Health Psychol. Off. J. Div. Health Psychol. Am. Psychol. Assoc.* **27**, 43–51 (2008).

28. Kramer, M. S. *et al.* Stress Pathways to Spontaneous Preterm Birth: The Role of Stressors, Psychological Distress, and Stress Hormones. *Am. J. Epidemiol.* **169**, 1319–1326 (2009).

29. Rose, M. S., Pana, G. & Premji, S. Prenatal Maternal Anxiety as a Risk Factor for Preterm Birth and the Effects of Heterogeneity on This Relationship: A Systematic Review and Meta-Analysis. *BioMed Res. Int.* **2016**, e8312158 (2016).

30. Phillips, G. S., Wise, L. A., Rich-Edwards, J. W., Stampfer, M. J. & Rosenberg, L. Prepregnancy Depressive Symptoms and Preterm Birth in the Black Women's Health Study. *Ann. Epidemiol.* **20**, 8–15 (2010).
31. Suri, R. *et al.* Effects of antenatal depression and antidepressant treatment on gestational age at birth and risk of preterm birth. *Am. J. Psychiatry* **164**, 1206–1213 (2007).
32. Barrios, Y., Sanchez, S., Qiu, C.-F., Gelaye, B. & Williams, M. Risk of spontaneous preterm birth in relation to maternal experience of serious life events during pregnancy. *Int. J. Womens Health* 249 (2014) doi:10.2147/IJWH.S54269.
33. Leeners, B., Stiller, R., Block, E., Görres, G. & Rath, W. Pregnancy complications in women with childhood sexual abuse experiences. *J. Psychosom. Res.* **69**, 503–510 (2010).
34. Sanchez, S. E. *et al.* Risk of Spontaneous Preterm Birth in Relation to Maternal Exposure to Intimate Partner Violence During Pregnancy in Peru. *Matern. Child Health J.* **17**, 485–492 (2013).
35. Lagdon, S., Armour, C. & Stringer, M. Adult experience of mental health outcomes as a result of intimate partner violence victimisation: a systematic review. *Eur. J. Psychotraumatology* **5**, 24794 (2014).
36. Miloyan, B., Joseph Bienvenu, O., Brilot, B. & Eaton, W. W. Adverse life events and the onset of anxiety disorders. *Psychiatry Res.* **259**, 488–492 (2018).
37. Sheng, J. A. *et al.* The Hypothalamic-Pituitary-Adrenal Axis: Development, Programming Actions of Hormones, and Maternal-Fetal Interactions. *Front. Behav. Neurosci.* **14**, (2021).
38. Magoun, H. W., Ranson, S. W. & Hetherington, A. Decending connections from the hypothalamus. *Arch. Neurol. Psychiatry* **39**, 1127 (1938).
39. Silverman, A. J., Hoffman, D. L. & Zimmerman, E. A. The descending afferent connections of the paraventricular nucleus of the hypothalamus (PVN). *Brain Res. Bull.* **6**, 47–61 (1981).
40. Dorton, A. The Pituitary Gland: Embryology, Physiology, and Pathophysiology. *Neonatal Netw.* **19**, 9–17 (2000).

41. Spencer, R. L. & Deak, T. A users guide to HPA axis research. *Physiol. Behav.* **178**, 43–65 (2017).

42. Jung, C. *et al.* A Longitudinal Study of Plasma and Urinary Cortisol in Pregnancy and Postpartum. *J. Clin. Endocrinol. Metab.* **96**, 1533–1540 (2011).

43. King, L. S., Humphreys, K. L., Cole, D. A. & Gotlib, I. H. Hair cortisol concentration across the peripartum period: Documenting changes and associations with depressive symptoms and recent adversity. *Compr. Psychoneuroendocrinology* **9**, 100102 (2022).

44. McLean, M. *et al.* A placental clock controlling the length of human pregnancy. *Nat. Med.* **1**, 460–463 (1995).

45. Bird, A. D., McDougall, A. R. A., Seow, B., Hooper, S. B. & Cole, T. J. Minireview: Glucocorticoid Regulation of Lung Development: Lessons Learned From Conditional GR Knockout Mice. *Mol. Endocrinol.* **29**, 158–171 (2015).

46. Liggins, G. C. The role of cortisol in preparing the fetus for birth. *Reprod. Fertil. Dev.* **6**, 141–150 (1994).

47. Stalder, T. & Kirschbaum, C. Analysis of cortisol in hair – State of the art and future directions. *Brain. Behav. Immun.* **26**, 1019–1029 (2012).

48. Raul, J.-S., Cirimele, V., Ludes, B. & Kintz, P. Detection of physiological concentrations of cortisol and cortisone in human hair. *Clin. Biochem.* **37**, 1105–1111 (2004).

49. Pragst, F. & Balikova, M. A. State of the art in hair analysis for detection of drug and alcohol abuse. *Clin. Chim. Acta* **370**, 17–49 (2006).

50. Wennig, R. Potential problems with the interpretation of hair analysis results. *Forensic Sci. Int.* **107**, 5–12 (2000).

51. Kirschbaum, C., Tietze, A., Skoluda, N. & Dettenborn, L. Hair as a retrospective calendar of cortisol production—Increased cortisol incorporation into hair in the third trimester of pregnancy. *Psychoneuroendocrinology* **34**, 32–37 (2009).

52. Khoury, J. E., Bosquet Enlow, M., Plamondon, A. & Lyons-Ruth, K. The association between adversity and hair cortisol levels in humans: A meta-analysis. *Psychoneuroendocrinology* **103**, 104–117 (2019).

53. Khoury, J. E. *et al.* Associations between psychological distress and hair cortisol during pregnancy and the early postpartum: A meta-analysis. *Psychoneuroendocrinology* **147**, 105969 (2023).

54. Duffy, A. R., Schminkey, D. L., Groer, M. W., Shelton, M. & Dutra, S. Comparison of Hair Cortisol Levels and Perceived Stress in Mothers Who Deliver at Preterm and Term. *Biol. Res. Nurs.* **20**, 292–299 (2018).

55. Gelaye, B. *et al.* Chronic HPA activity in mothers with preterm delivery: A pilot nested case-control study. *J. Neonatal-Perinat. Med.* **13**, 313–321 (2020).

56. Hoffman, M. C., Mazzoni, S. E., Wagner, B. D., Laudenslager, M. L. & Ross, R. G. Measures of Maternal Stress and Mood in Relation to Preterm Birth. *Obstet. Gynecol.* **127**, 545–552 (2016).

57. Karakash, S. D. *et al.* Hypocortisolism and preterm birth. *J. Neonatal-Perinat. Med.* **9**, 333–339 (2016).

58. Musana, J. *et al.* Obstetric risk in pregnancy interacts with hair cortisone levels to reduce gestational length. *Front. Glob. Womens Health* **3**, 878538 (2022).

59. Schonblum, A. *et al.* Can hair steroids predict pregnancy longevity? *Reprod. Biol.* **18**, 410–415 (2018).

60. Stoye, D. Q. *et al.* Perinatal determinants of neonatal hair glucocorticoid concentrations. *Psychoneuroendocrinology* **128**, 105223 (2021).

61. Steudte-Schmiedgen, S., Kirschbaum, C., Alexander, N. & Stalder, T. An integrative model linking traumatization, cortisol dysregulation and posttraumatic stress disorder: Insight from recent hair cortisol findings. *Neurosci. Biobehav. Rev.* **69**, 124–135 (2016).

62. Barrios, Y. V. *et al.* Childhood Abuse and Early Menarche Among Peruvian Women. *J. Adolesc. Health* **56**, 197–202 (2015).
63. ACOG. ACOG technical bulletin. Preterm labor. Number 206--June 1995 (Replaces No. 133, October 1989). *Int. J. Gynaecol. Obstet. Off. Organ Int. Fed. Gynaecol. Obstet.* **50**, 303–313 (1995).
64. Orta, O. R. *et al.* An evaluation of distal hair cortisol concentrations collected at delivery. *Stress* **21**, 355–365 (2018).
65. Buse, J. *et al.* Hair cortisol-a stress marker in children and adolescents with chronic tic disorders? A large European cross-sectional study. *Eur. Child Adolesc. Psychiatry* **31**, 771–779 (2022).
66. Dong, S. *et al.* Maternal exposure to black carbon and nitrogen dioxide during pregnancy and birth weight: Using machine-learning methods to achieve balance in inverse-probability weights. *Environ. Res.* **211**, 112978 (2022).
67. Wu, X., Mealli, F., Kioumourtzoglou, M.-A., Dominici, F. & Braun, D. Matching on Generalized Propensity Scores with Continuous Exposures. *J. Am. Stat. Assoc.* (2022).
68. Cripe, S. M. *et al.* Association of intimate partner physical and sexual violence with unintended pregnancy among pregnant women in Peru. *Int. J. Gynecol. Obstet.* **100**, 104–108 (2008).
69. Perales, M. T. *et al.* Prevalence, Types, and Pattern of Intimate Partner Violence Among Pregnant Women in Lima, Peru. *Violence Women* **15**, 224–250 (2009).
70. Zhong, Q.-Y. *et al.* Childhood abuse and suicidal ideation in a cohort of pregnant Peruvian women. *Am. J. Obstet. Gynecol.* **215**, 501.e1-501.e8 (2016).
71. Marceau, K., Wang, W., Robertson, O. & Shirtcliff, E. A. A systematic review of hair cortisol during pregnancy: Reference ranges and methodological considerations. *Psychoneuroendocrinology* **122**, 104904 (2020).

72. Kelsall, N. C. *et al.* Association between trauma exposure and glucocorticosteroid concentration in hair during pregnancy. *Psychoneuroendocrinology* **151**, 106072 (2023).
73. Schalinski, I., Elbert, T., Steudte-Schmiedgen, S. & Kirschbaum, C. The Cortisol Paradox of Trauma-Related Disorders: Lower Phasic Responses but Higher Tonic Levels of Cortisol Are Associated with Sexual Abuse in Childhood. *PLOS ONE* **10**, e0136921 (2015).
74. Wichmann, S., Kirschbaum, C., Böhme, C. & Petrowski, K. Cortisol stress response in post-traumatic stress disorder, panic disorder, and major depressive disorder patients. *Psychoneuroendocrinology* **83**, 135–141 (2017).
75. Suzuki, A., Poon, L., Papadopoulos, A. S., Kumari, V. & Cleare, A. J. Long term effects of childhood trauma on cortisol stress reactivity in adulthood and relationship to the occurrence of depression. *Psychoneuroendocrinology* **50**, 289–299 (2014).
76. Steudte-Schmiedgen, S. *et al.* Hair cortisol concentrations and cortisol stress reactivity predict PTSD symptom increase after trauma exposure during military deployment. *Psychoneuroendocrinology* **59**, 123–133 (2015).
77. Elzinga, B. M., Schmahl, C. G., Vermetten, E., Van Dyck, R. & Bremner, J. D. Higher Cortisol Levels Following Exposure to Traumatic Reminders in Abuse-Related PTSD. *Neuropsychopharmacology* **28**, 1656–1665 (2003).
78. Gola, H. *et al.* Victims of rape show increased cortisol responses to trauma reminders: A study in individuals with war- and torture-related PTSD. *Psychoneuroendocrinology* **37**, 213–220 (2012).

### 9 Supplementary Material

**Table S1**. Missingness in selected covariates (*N*=1,808)

| Covariates | Number of missingness (%) |
|---|---|
| Maternal age | 1 (0.1) |
| Difficulty accessing basic foods | 9 (0.5) |
| Employment during pregnancy | 2 (0.1) |
| Marital status | 5 (0.3) |
| Nulliparity | 6 (0.3) |
| Race | 3 (0.2) |
| Planned pregnancy | 11 (0.6) |
| Gestational age at hair collection | 1 (0.1) |
| Infant sex | 32 (1.8) |
| Hair dye | 26 (1.4) |
| Hair tint | 25 (1.4) |
| Hair wash | 24 (1.3) |
| Pre-pregnant BMI | 10 (0.6) |
| BMI at pregnancy | 14 (0.8) |

**Appendix S1.** Description of multiple imputation

The imputations for missing covariates were conducted among participants with complete records of hair corticosteroid concentration and status of preterm birth. Multivariate Imputation by Chained Equation (MICE) was applied separately when assessing different indicators of corticosteroids. All variables in this study, including the outcome (i.e., preterm birth), were included as the predictors for MICE to minimize the bias caused by the missingness of covariates. For imputed analysis for each indicator of corticosteroids, the number of imputed datasets (m) was determined by the formula: $Relative\ efficiency\ (RE) = \left(1 + \frac{\lambda}{m}\right)^{-1}$, where lambda is the proportion of incomplete cases, and RE was set to be 95%. The m was rounded up to the nearest integer. For each imputed dataset, the iteration number was 20. The results of the imputation show satisfied convergence. In each imputed dataset, we reran the primary analysis before pooling the estimates using the *miceadd* package in R.

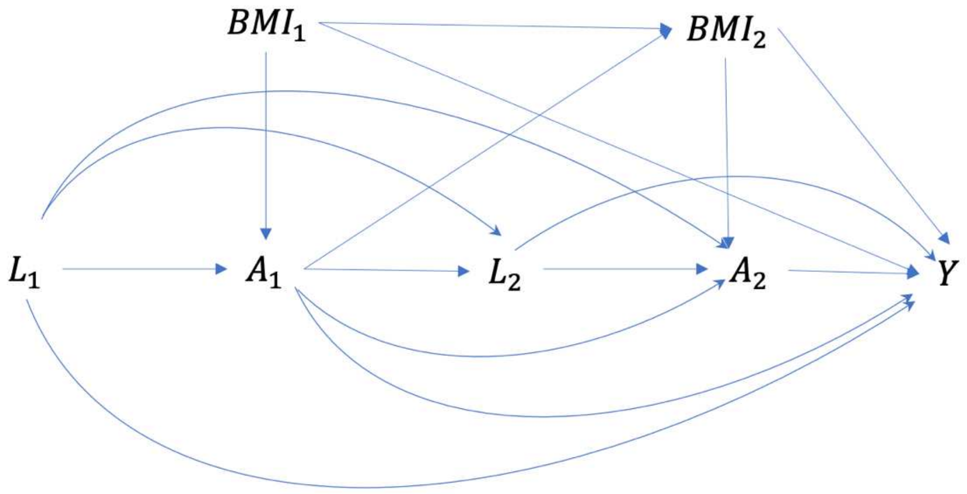

**Fig. S1**. The directed acyclic graph (DAG) describing the association among hair corticosteroids concentration, preterm birth, and selected covariates

$A_1$: Hair corticosteroids concentration 0-3 months before pregnancy

$A_2$: Hair corticosteroids concentration in the first trimester of pregnancy

$L_1$: Maternal age, difficulty accessing basic foods, race, marital status, parity, gestational age at hair collection, hair treatment (hair tint, dye, and washing frequency)

$L_2$: Employment during pregnancy, planned pregnancy, infant sex

$BMI_1$: Body mass index (BMI) before pregnancy

$BMI_2$: Body mass index (BMI) in the first trimester of pregnancy

Y: Preterm birth

**Table S2.** Variables used to construct the propensity score model for each occasion of hair corticosteroid concentration and formula of SIPW

| Exposure ($A$) | Potential confounders ($C$) | Exposure in the last occasion ($A$) | SIPW |
|---|---|---|---|
| Pre-pregnancy ($A_1$) | Maternal age, difficulty accessing basic foods, race, marital status, parity, gestational age at hair collection, hair treatment (hair tint, dye, and washing frequency), and body mass index (BMI) before pregnancy | - | $\frac{f_{A_1}(A_1; \mu_x, \sigma_x^2)}{f_{A_1}(A_1 \mid C = c; \mu_y, \sigma_y^2)}$ |
| First trimester ($A_2$) | Maternal age, difficulty accessing basic foods, race, marital status, parity, gestational age at hair collection, hair treatment (hair tint, dye, and washing frequency), employment during pregnancy, planned pregnancy, infant sex, and body mass index (BMI) in the first trimester of pregnancy | Pre-pregnancy ($A_1$) | $\frac{f_{A_2}(A_2 \mid A_1 = a_1; \mu_x, \sigma_x^2)}{f_{A_2}(A_2 \mid C = c, A_1 = a_1; \mu_y, \sigma_y^2)}$ |

**Table S3.** Marginal association between hair corticosteroid levels and risk of preterm birth after multiple imputation

| | Pre-pregnancy | First trimester |
|---|---|---|
| | RR (95%CI) | RR (95%CI) |
| Log HCC[a] (*N* = 1739) | 0.91 (0.76,1.11) | 1.41 (1.16,1.71) |
| Log HCNC (*N* = 1609) | 0.84 (0.61,1.16) | 1.23 (0.93,1.65) |

Abbreviation: HCC, hair cortisol concentration; HCNC, hair cortisone concentration.
[a] Log HCC and Log HCNC have been standardized (mean=0 and SD=1).

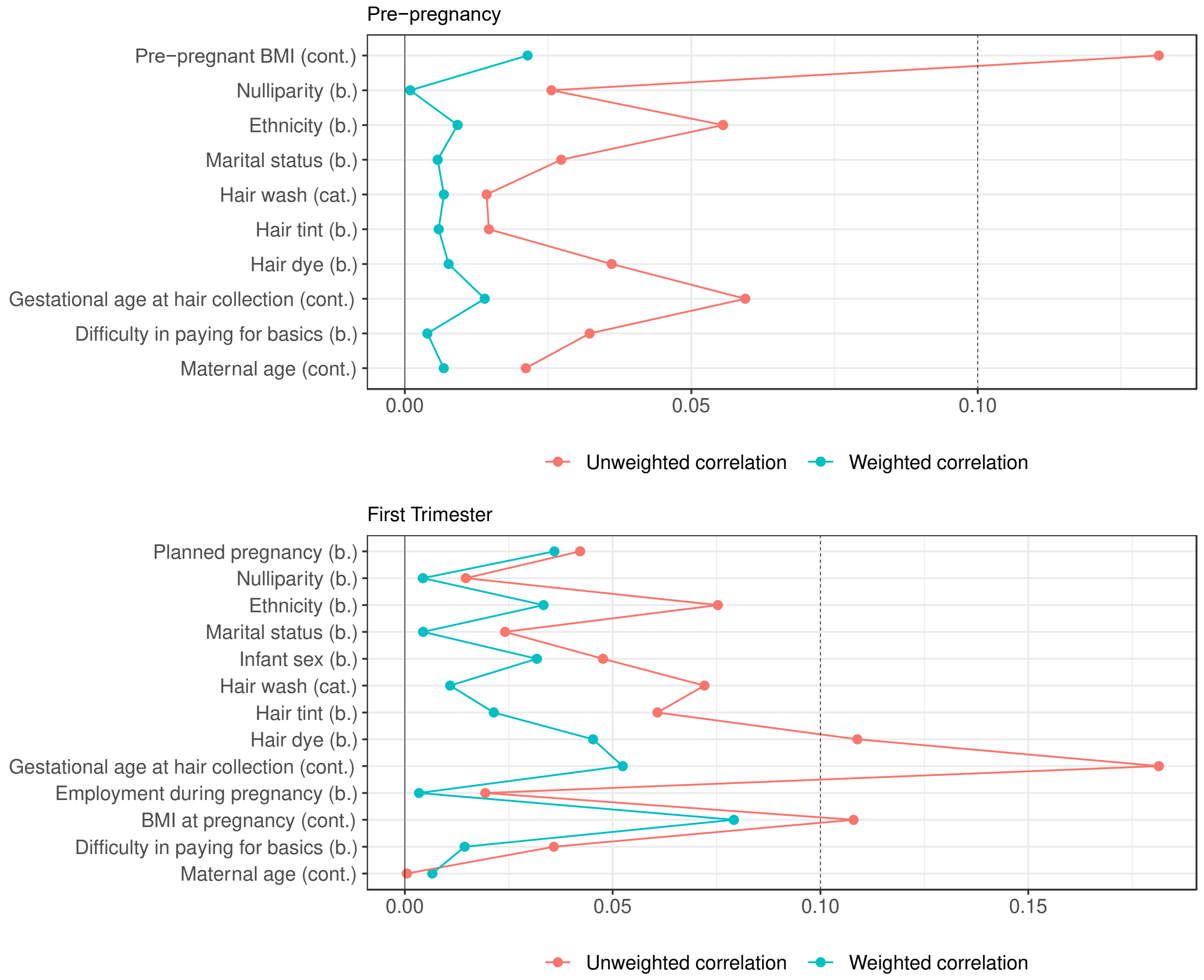

**Fig. S2.** Absolute correlation between Log HCC on different occasions and each potential confounder with and without SIPW.

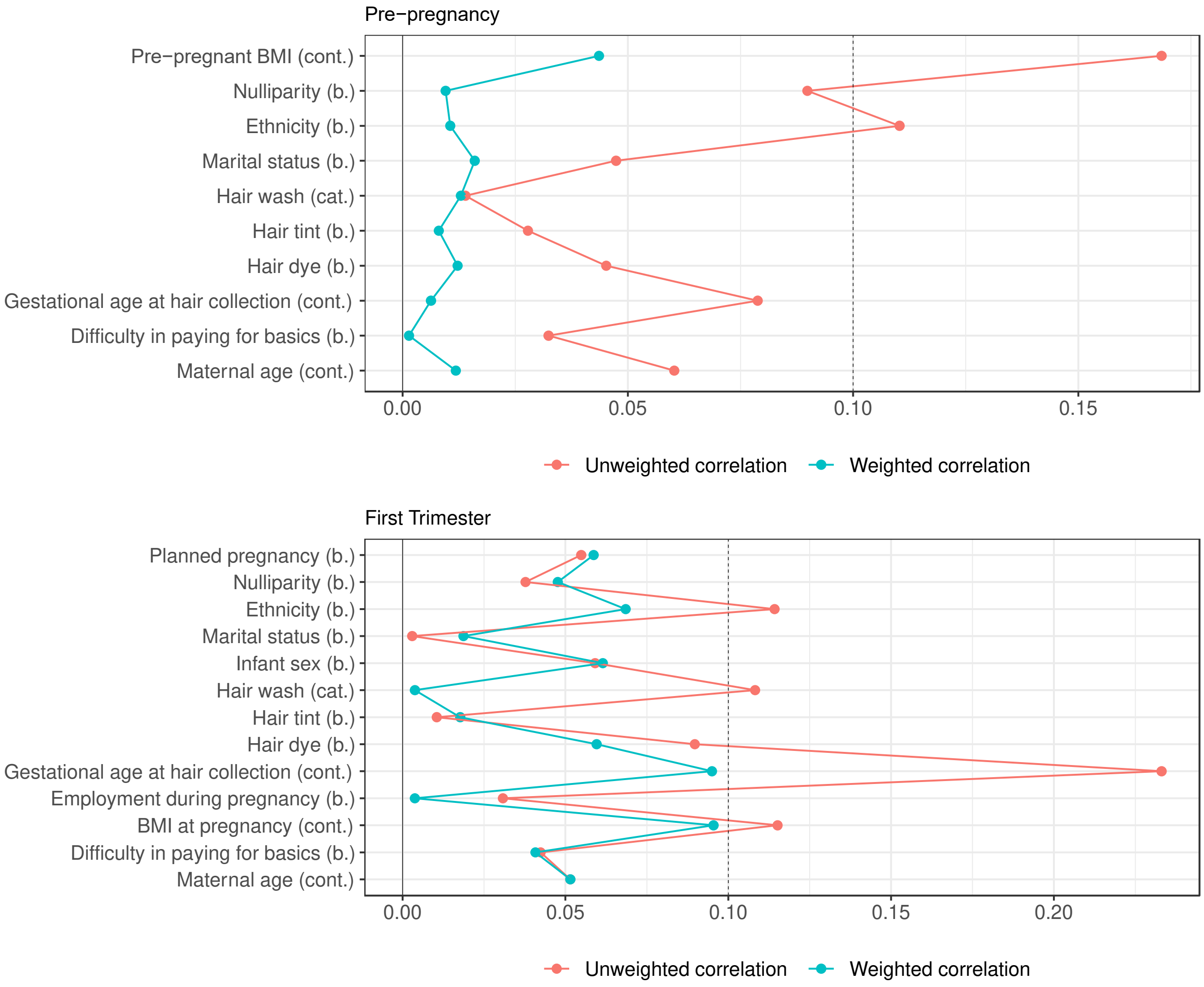

**Fig. S3.** Absolute correlation between Log HCNC on different occasions and each potential confounder with and without SIPW.

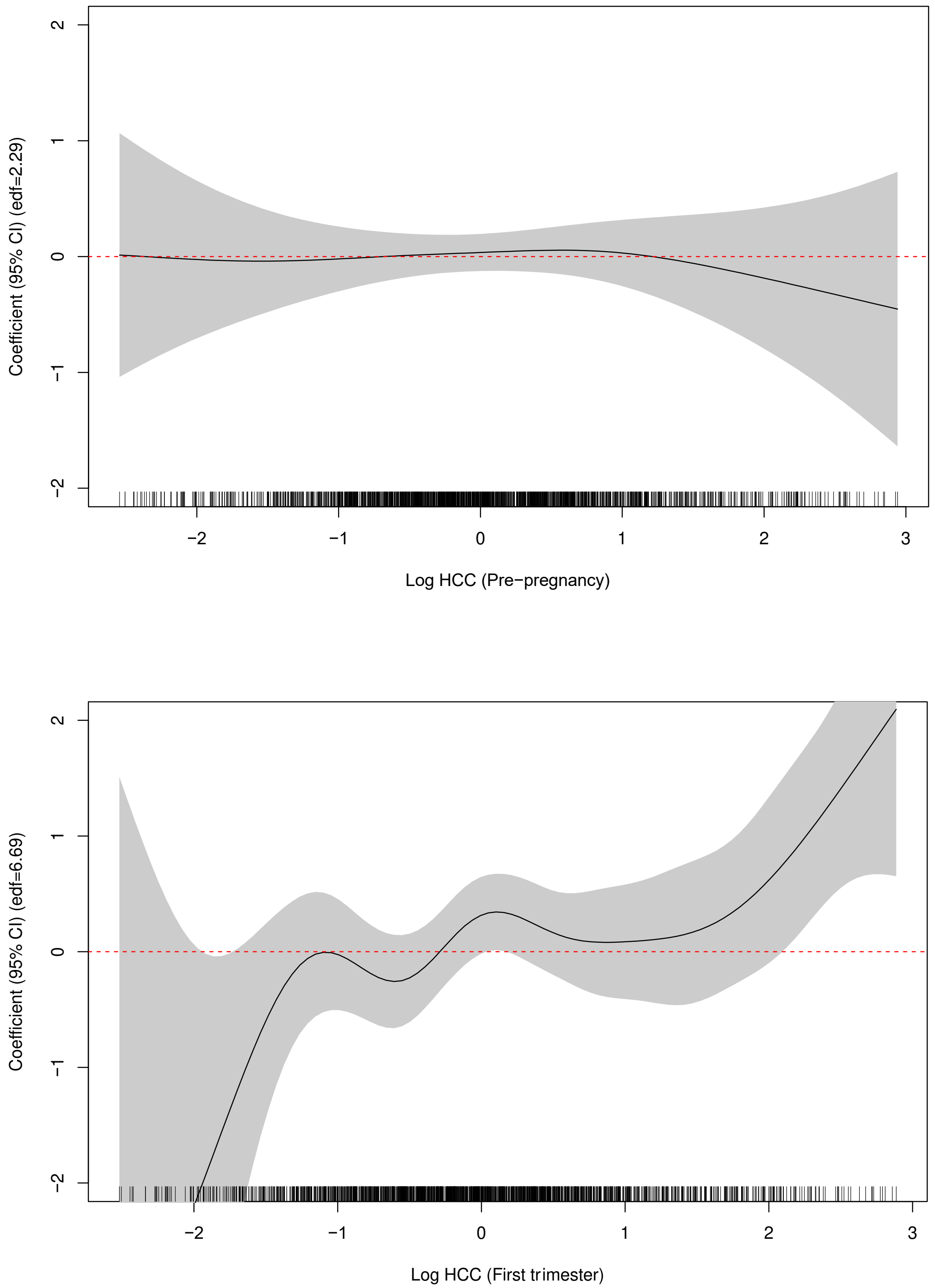

**Fig. S4.** Curvilinear pattern of the association between HCC and preterm birth risk (in Log scale)
**Abbreviation:** edf = effective degrees of freedom
**Note:** P for non-linearity = 0.13

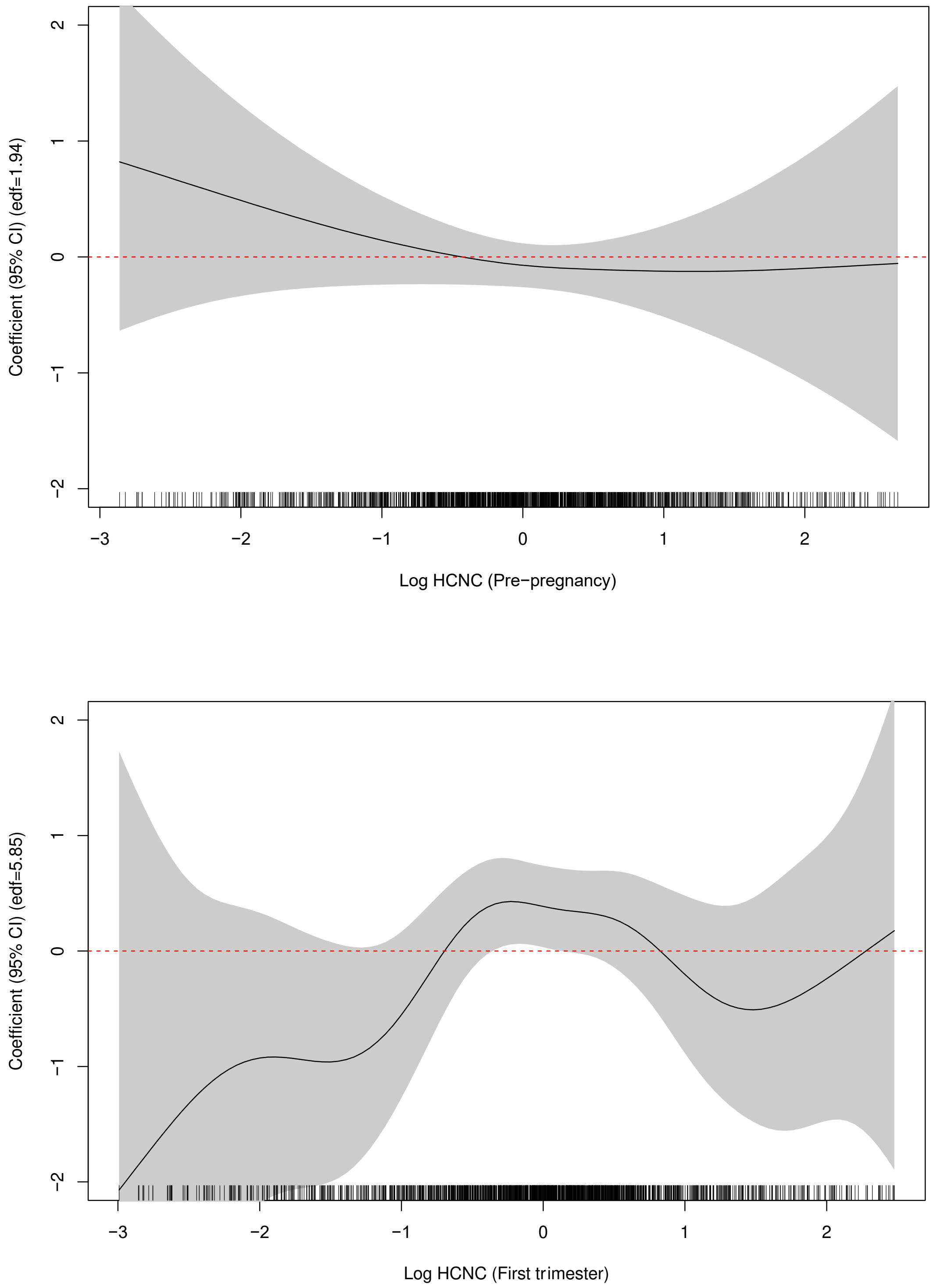

**Fig. S5.** Curvilinear pattern of the association between HCNC and preterm birth risk (in Log scale)
**Abbreviation:** edf = effective degrees of freedom
**Note:** P for non-linearity = 0.07

**Table S4.** Marginal association between hair corticosteroid levels and risk of preterm birth (using multivariate linear regression to estimate propensity score)

| | Pre-pregnancy | First trimester |
|---|---|---|
| | RR (95%CI) | RR (95%CI) |
| Log HCC[a] (*N* = 1647) | 0.95 (0.77,1.16) | 1.39 (1.13,1.71) |
| Log HCNC (*N* = 1520) | 0.84 (0.58,1.20) | 1.20 (0.87,1.65) |

Abbreviation: HCC, hair cortisol concentration; HCNC, hair cortisone concentration

[a] Log HCC and Log HCNC have been standardized (mean=0 and SD=1).

**Table S5.** Weighted average absolute correlation between hair corticosteroid levels and each covariate using different approaches

| | Pre-pregnancy | | First trimester | |
|---|---|---|---|---|
| | Weighted AAC (GAM) | Weighted AAC (LM) | Unweighted AAC (GAM) | Weighted AAC (LM) |
| Log HCC (*N* = 1647) | 0.008 | 0.008 | 0.026 | 0.025 |
| Log HCNC (*N* = 1520) | 0.013 | 0.013 | 0.048 | 0.046 |

Abbreviation: AAC, average absolute correlation; GAM, generalized additive model; HCC, hair cortisol concentration; HCNC, hair cortisone concentration; LM, linear regression

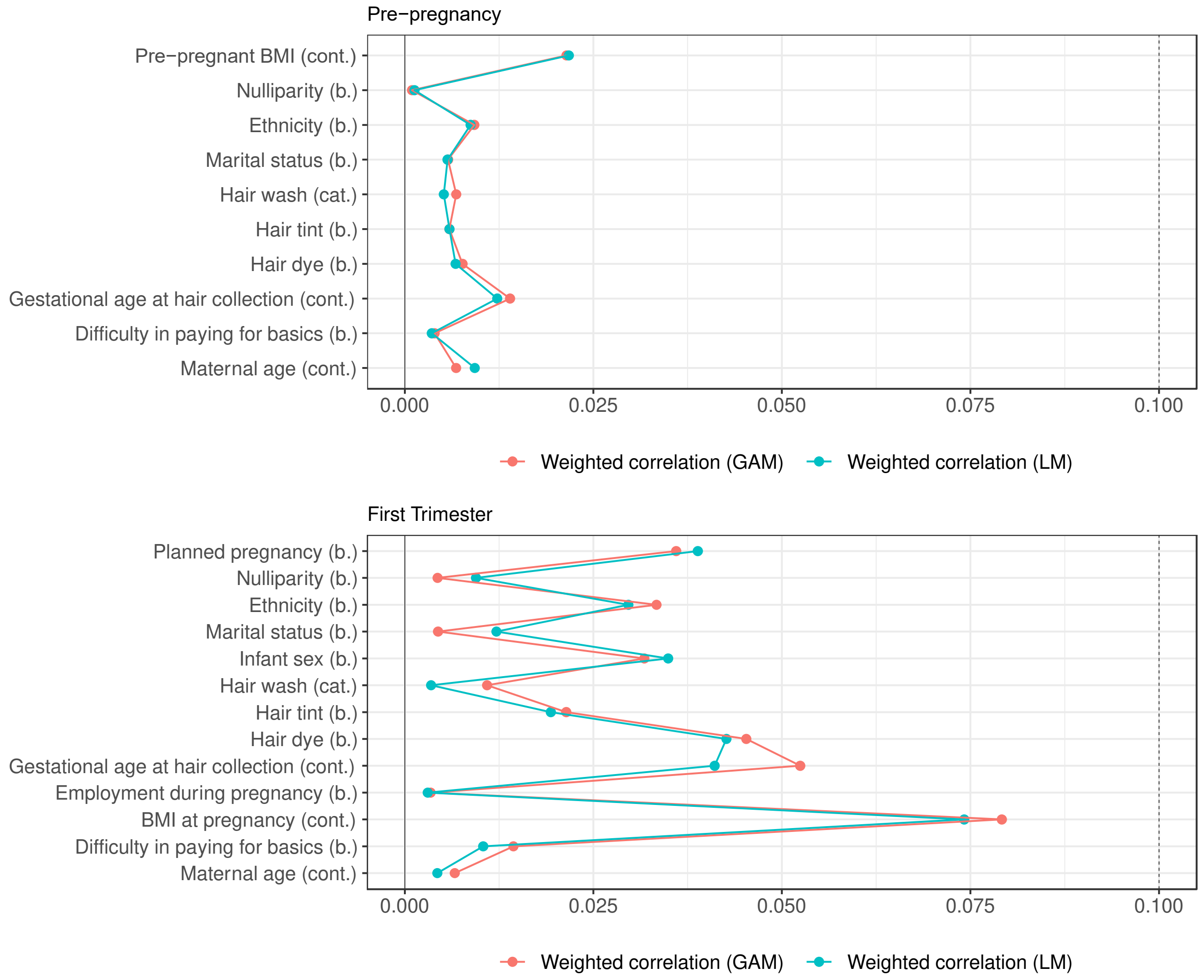

**Fig. S6.** Pairwise weighted absolute correlation between Log HCC on different occasions and each potential confounder by different approaches

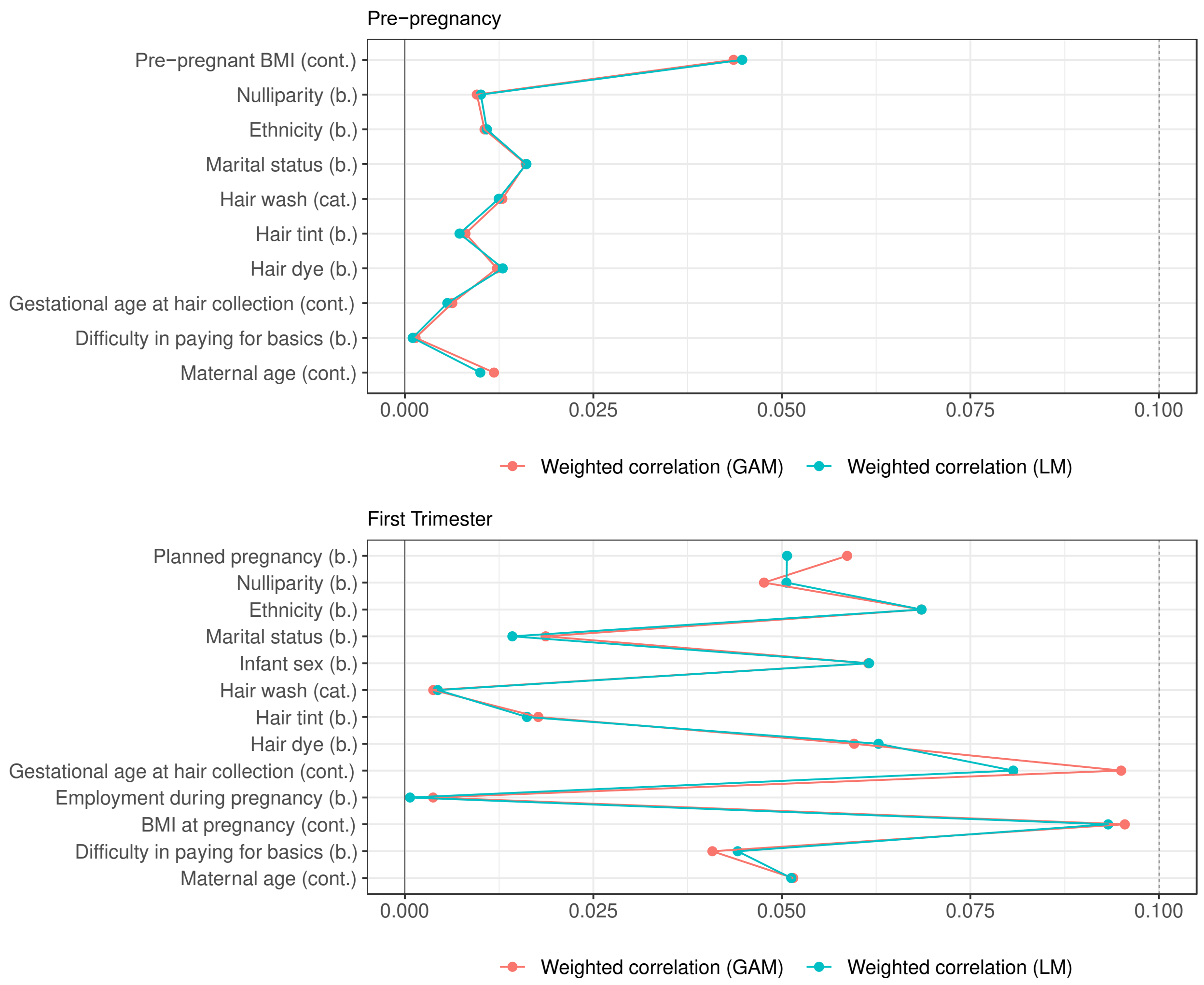

**Fig. S7.** Pairwise weighted absolute correlation between Log HCNC on different occasions and each potential confounder by different approaches
